# Reducing dementia risk by targeting modifiable risk factors in mid-life: study protocol for the Innovative Midlife Intervention for Dementia Deterrence (In-MINDD) randomised controlled feasibility trial

**DOI:** 10.1186/s40814-015-0035-x

**Published:** 2015-11-17

**Authors:** Catherine A. O’Donnell, Susan Browne, Maria Pierce, Alex McConnachie, Kay Deckers, Martin P. J. van Boxtel, Valeria Manera, Sebastian Köhler, Muriel Redmond, Frans R. J. Verhey, Marjan van den Akker, Kevin Power, Kate Irving, In-MINDD Team

**Affiliations:** 1 General Practice and Primary Care, Institute of Health & Wellbeing, University of Glasgow, 1 Horselethill Road, Glasgow, G12 9LX Scotland UK; 2 School of Nursing and Human Sciences, Dublin City University, Dublin, Ireland; 3 Robertson Centre for Biostatistics, Institute of Health & Wellbeing, University of Glasgow, Glasgow, UK; 4 School for Mental Health and Neuroscience, Alzheimer Centrum Limburg, Maastricht University, Maastricht, The Netherlands; 5 CoBTeK COgnition Behaviour Technology, Université de Nice Sophia Antipolis, Nice, France

**Keywords:** Dementia, Primary prevention, Modifiable risk factors, Internet, Primary care

## Abstract

**Background:**

Dementia prevalence is increasing as populations live longer, with no cure and the costs of caring exceeding many other conditions. There is increasing evidence for modifiable risk factors which, if addressed in mid-life, can reduce the risk of developing dementia in later life. These include physical inactivity, low cognitive activity, mid-life obesity, high blood pressure, and high cholesterol. This study aims to assess the acceptability and feasibility and impact of giving those in mid-life, aged between 40 and 60 years, an individualised dementia risk modification score and profile and access to personalised on-line health information and goal setting in order to support the behaviour change required to reduce such dementia risk. A secondary aim is to understand participants’ and practitioners’ views of dementia prevention and explore the acceptability and integration of the Innovative Midlife Intervention for Dementia Deterrence (In-MINDD) intervention into daily life and routine practice.

**Methods/design:**

In-MINDD is a multi-centre, primary care-based, single-blinded randomised controlled feasibility trial currently being conducted in four European countries (France, Ireland, the Netherlands and the UK). Participants are being recruited from participating general practices. Inclusion criteria will include age between 40 and 60 years; at least one modifiable risk factor for dementia risk (including diabetes, hypertension, obesity, renal dysfunction, current smoker, raised cholesterol, coronary heart disease, current or previous history of depression, self-reported sedentary lifestyle, and self-reported low cognitive activity) access to the Internet. Primary outcome measure will be a change in dementia risk modification score over the timescale of the trial (6 months). A qualitative process evaluation will interview a sample of participants and practitioners about their views on the acceptability and feasibility of the trial and the links between modifiable risk factors and dementia prevention. This work will be underpinned by Normalisation Process Theory.

**Discussion:**

This study will explore the feasibility and acceptability of a risk profiler and on-line support environment to help individuals in mid-life assess their risk of developing dementia in later life and to take steps to alleviate that risk by tackling health-related behaviour change. Testing the intervention in a robust and theoretically informed manner will inform the development of a future, full-scale randomised controlled trial.

**Trial registration:**

ISRCTN Registry: ISRCTN 98553005 (DOI: 10.1186/ISRCTN98553005).

**Electronic supplementary material:**

The online version of this article (doi:10.1186/s40814-015-0035-x) contains supplementary material, which is available to authorized users.

## Background

Dementia is a serious loss of cognitive ability beyond what might be expected from normal ageing, with Alzheimer’s disease and vascular dementia, the commonest types [1]. Current estimates suggest a worldwide prevalence of dementia in those aged 60 and over at 5 to 7 %, with numbers increasing from 35.6 million in 2010 to an estimated 115.4 million in 2050 [2, 3]. In 2010, the global cost of dementia and the associated needs for care were put at £391 billion (€533 billion, US $604 billion) [1]. In the UK, for example, dementia costs the economy £17 billion (€23 billion, $26 billion) per annum—more than cancer and heart disease combined [4]. In addition to the economic and caring burdens, dementia is a condition that arouses fear and uncertainty. Currently incurable, the onset and development of dementia can create an enormous sense of insecurity for individuals and their families. Research has demonstrated high levels of anxiety amongst middle-aged and young-old individuals about their memory. This is compounded by the debilitation that can be associated with late-stage dementia, making it one of the most feared conditions in relation to ageing [5, 6]. Taken together, there is a clear need to not only develop treatments for those with dementia, but to develop our understanding of the ways in which dementia risk might addressed and reduced in those still in mid-life, well before the onset of dementia. This is the aim of Innovative Midlife Intervention for Dementia Deterrence (In-MINDD).

### Risk factors associated with dementia

Several risk factors have been identified which can either augment or reduce one’s risk of developing dementia [6, 7]. Some are non-modifiable, in particular age and genetic factors such as apolipoprotein Ɛ4 [8]. An increasing body of evidence is, however, highlighting a role for modifiable risk factors which exacerbate, or reduce, one’s risk of developing dementia in later life. Systematic reviews of observational studies and randomised controlled trials have examined the evidence for the influence of a range of modifiable factors in later cognitive decline and dementia [9–11]. Kloppenborg et al. found that diabetes, hypertension, high cholesterol and obesity were each associated with an increased risk of dementia, although the evidence was most consistent for diabetes and obesity [9]. Plassman and colleagues identified a range of potential risk factors: depression, type 2 diabetes and smoking increasing the risk; vegetable intake, Mediterranean diet, increased physical and cognitive activity ameliorating it [10]. However in general, the evidence identified was of low quality and derived mainly from observational studies. A later review calculated the population attributable risk associated with seven modifiable risk factors (diabetes, midlife hypertension, midlife obesity, smoking, depression, physical inactivity, and low educational attainment) [11]. As well as finding that each risk factor alone increased the relative risk of developing dementia in later life, the authors also calculated that these seven risk factors together accounted for approximately 50 % of all cases of Alzheimer’s dementia.

Two more recent papers have developed this evidence base. Deckers et al. combined a systematic review of observational studies with a Delphi study which asked international experts in the field of dementia prevention and epidemiology to rank and weight the identified risk factors [12]. This study identified depression, midlife obesity, high cholesterol, midlife hypertension, diabetes, physical inactivity, and smoking as associated with an increased risk of developing dementia in later life. Evidence relating to diet, cognitive activity, coronary heart disease and renal dysfunction was, however, inconclusive.

One criticism of some of this work has been the failure to account for the interdependence of many of these risk factors [13]. Thus, one recent paper has calculated the population attributable risk for the seven risk factors previously identified by Barnes and Yaffe [11]: the authors calculated the individual risk, the combined risk and, elegantly, the combined risk accounting for the interdependence of many of the risk factors (e.g. midlife obesity, physical inactivity and low educational attainment) [14]. Even accounting for the inter-relationship of risk factors, approximately 30 % of Alzheimer’s cases worldwide can be attributed to the aforementioned risk factors of diabetes, midlife hypertension, midlife obesity, smoking, depression, physical inactivity, and low educational attainment.

This suggests that interventions targeting those in mid-life to address health-related behaviour change around modifiable risk factors might reduce both individuals’ risk of developing dementia in later life and, as a consequence, the prevalence of dementia [11, 14, 15]. There is, however, a lack of public awareness about the link between such modifiable risk factors and dementia risk [16, 17]. In addition, even if such links are made, such behaviour change is not always easy.

### Making and sustaining health behaviour change

Making and sustaining health behaviour changes, such as stopping smoking or increasing physical activity, is not easy [18, 19]. Interventions that utilise the Internet and social media are increasingly seen as one potential approach to empowering and sustaining individuals trying to make and maintain such changes to their daily routine [20–23]. Systematic reviews indicate that web-based and eHealth interventions can be effective in supporting behaviour change [24]. For example, web-based interventions targeting smoking cessation [25] or increasing physical activity [26, 27] are effective in promoting, at least in the short-term, the desired change in behaviour. In other areas, however, the evidence is more equivocal, e.g. in reducing alcohol consumption [28] or changing dietary patterns [29]. There are similarly positive and negative findings where multiple behaviours are targeted [30–32]. This variability in the findings may be due, in part, to the quality of the underlying evidence base [31] or to a lack of consideration of what components of the intervention are the “active ingredients” [24]. Nevertheless, the potential to scale up even moderately effective interventions to reach large populations suggest that web-based interventions are worth developing and testing.

There are, however, well-recognised problems associated with scaling up the implementation of such approaches, particularly in relation to sustainability and the embedding of such approaches in both the daily lives of patients and in the clinical routine of practitioners [33–36]. To address these issues, Murray suggests that more attention to be paid to early intervention development and testing and to more explicit use of theory to inform the development of and understand how interventions are embedded and routinized by users [24]. This use of theory is supported by Webb et al., who found that theoretically developed web-based interventions were more effective at supporting behaviour change [27]. It is these considerations which have underpinned the methodological design of the In-MINDD randomised controlled feasibility trial.

### The Innovative Midlife Intervention for Dementia Deterrence study

Funded by the EU Framework 7 programme, In-MINDD brings together these two important concepts: first, that there is a group of potentially modifiable and inter-linked risk factors which, if addressed in mid-life, may reduce the risk of developing dementia in later life, or at least delay its onset. Second, that on-line interactive tools may help individuals make and—importantly—sustain health-related behavioural change. Earlier work in In-MINDD identified a group of modifiable risk factors and conditions associated with dementia risk (Table 1) [12]. Using the relative risks from the identified literature, the In-MINDD team developed a risk score algorithm in which the relative risk of each factor was standardised and weighted to a reference value, in this case, the relative risk for low/moderate alcohol consumption. The final model, based on the 12 risk factors shown in Table 1, is then used to produce a personalised lifestyle for brain health (LIBRA) global score and profile for individuals participating in the feasibility trial (manuscript in preparation, Schiepers et al.).

**Table 1:** Modifiable risk factors and conditions identified by In-MINDD as potentially increasing or reducing dementia risk (adapted from [12])

Risk/protective factor	Relative risk from published literature	Weight applied to factor for LIBRA global score
Low/moderate alcohol consumption	0.74	−1.0
Coronary heart disease	1.36	+1.0
Physical inactivity	1.39	+1.1
Renal dysfunction	1.39	+1.1
Diabetes	1.47	+1.3
Raised cholesterol level	1.54	+1.4
Smoking	1.59	+1.5
(Midlife) obesity	1.60	+1.6
(Midlife) hypertension	1.61	+1.6
Mediterranean diet	0.60	−1.7
Depression	1.85	+2.1
Cognitive activity	0.38	−3.2

**Fig. 1:**
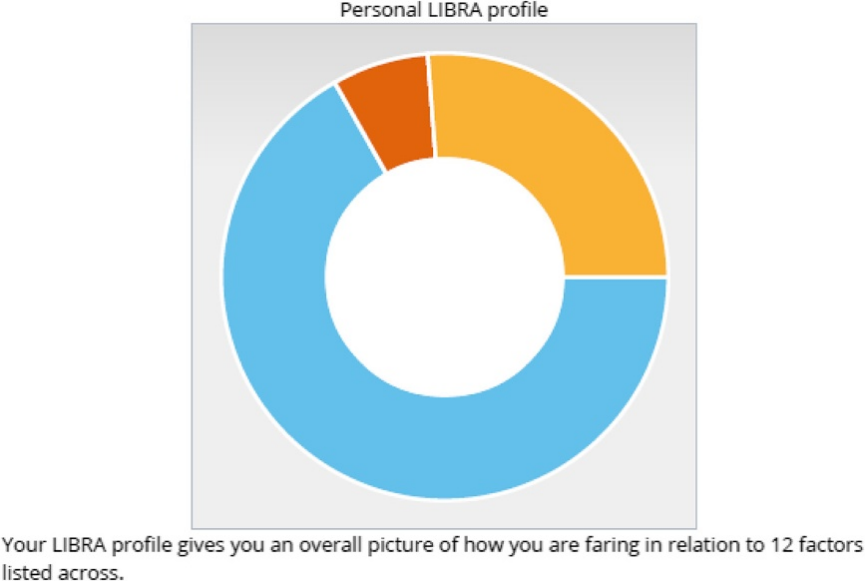
This LIBRA profile give a participant the following information. Blue segment represents their “Keep This Up” score of 67 %. The participant is told those risk factors which they are currently managing well (in this example, cholesterol level; cognitive activity; alcohol consumption; mood; physical activity; and smoking) or conditions which they currently do not have (heart disease; chronic kidney disease). Amber section represents their “Room for Improvement” score of 26 %. This is made up of blood pressure; diet; and obesity. These are areas which would be targeted for behaviour change strategies. Dark red section represents their “Remember to Manage Well” score of 7 %. This is due the participant having diabetes. See Table 1 for a breakdown of contributing risk factors.

From this, we have developed an on-line profiler and support environment which, based on individualised demographic, clinical and self-reported information on health-related behaviours, can calculate an individual’s dementia risk modification score. This information is given to individuals as a personalised LIBRA global score and profile, highlighting areas of health-related behaviour in which they are doing well (e.g. if they are a non-smoker); areas which they cannot change but need to manage (e.g. if they have diabetes) and areas where they could make improvements (e.g. by increasing physical activity). This is illustrated in Fig. 1.

In addition to the LIBRA score and profile, there is a personalised on-line support environment which supports goal setting and gives access to health information (manuscript in preparation by In-MINDD team).

In-MINDD is now testing the feasibility and acceptability of the LIBRA score and profile and on-line support environment in primary care across four European countries: France, Ireland, the Netherlands and the UK (namely Scotland). Primary care has been selected as the setting as this affords an opportunity to ensure that primary care practitioners, as well as patients, are informed of the existence of the identified risk factors especially as such factors are widely prevalent in the European population [37]. It also gives GPs an opportunity to support and motivate patients as they make, and hopefully sustain, health-related behaviour change.

In order to do this, we are conducting a theoretically informed feasibility randomised controlled trial and process evaluation.

### Rationale for the methodological approach

The MRC framework for complex interventions recommends that before full-scale randomised controlled trials are conducted, all stages of a trial should be fully piloted and the feasibility of the trial approach evaluated [38, 39]. A feasibility study is designed, not to measure effectiveness, but to assess the various components of a trial in order to ensure that it is acceptable and feasible when scaled up [40–42]. In particular, a feasibility trial is required when there are uncertainties about issues such as identification of participants, recruitment and retention, acceptability of the overall intervention or the outcome measure on which to base a sample size calculation.

Another key consideration for optimising the learning from a trial is having an underpinning theoretical framework [24]. As described previously, the advantages of theory in the development of a complex intervention such as this are well reported. Theory can provide a framework that is generalizable across settings and individuals; it provides the opportunity for the incremental generation of knowledge and provides a framework to guide analysis [43, 44]. It can also highlight and enhance our understanding of the barriers to implementation and alert us to the context into which new interventions and services are placed [39, 45–47].

This is particularly important for a research project such as In-MINDD, where it is crucial to understand and evaluate issues such as participants’ understanding of dementia prevention, the use of the on-line profiler and support environment, as well as to test and improve the various stages of the trial itself such as identification, recruitment and retention of participants. In order to fully understand the barriers and facilitators to implementing and using the In-MINDD intervention in participants’ lives and in routine general practice, we need a theoretical approach which allows us to understand the work involved in implementing such a complex intervention. The theory selected is Normalisation Process Theory (NPT).

NPT is a mid-range sociological theory concerned with the work that individuals and organisations have to carry out in order to embed and normalise new, complex ways of working into daily routine practice [48, 49]. NPT alerts researchers and implementers alike to the realities of implementation in real time and the interactions that do, or do not, occur between the individuals and groups charged with that implementation [46, 50]. NPT does this by focusing attention on four broad areas, described by four constructs, which are important in the implementation of new ways of working (Table 2).

**Table 2:** NPT constructs

Construct	What it addresses
Coherence	Can those involved in the implementation make sense of it?
Cognitive participation	Can those involved in the implementation maintain their involvement and get others involved and engaged?
Collective action	What has to be done to make the intervention being implemented work in routine practice?
Reflexive monitoring	How can the intervention be monitored and evaluated? Can it be re-designed?

NPT will thus be used to guide the qualitative process evaluation, when both participants and practitioners will be interviewed about their experience and views of the In-MINDD profiler, the LIBRA global score and profile and the on-line support environment. These questions are important to In-MINDD as we seek to understand how much participants and practitioners know about the links between modifiable risk factors and dementia risk; whether that knowledge increases the chance of participants making and sustaining health-related behaviour change; and whether the In-MINDD profiler and support environment supports them in that behaviour change.

### Study aims

The aim of the In-MINDD feasibility RCT is to assess the acceptability and feasibility of giving those in mid-life, aged between 40 and 60 years, an individualised LIBRA brain health score and profile and access to personalised on-line health information designed to support health-related behaviour change. Secondly, the study aims to understand participants’ views of dementia prevention and explore the acceptability and integration of the In-MINDD intervention into daily life and routine practice. This information will be used to design and develop a definitive trial. Finally, we aim to collect information on patient-centred outcomes to determine if a change in behaviour can be detected (i.e. proof of concept) and determine the most appropriate main outcome measure for the main trial.

The study will thus collect a range of quantitative and qualitative data from participants and from primary care practitioners in four European primary care systems.

## Design and methods

### Study design

This is a multi-centre, primary care-based, randomised controlled feasibility trial currently being conducted in four European countries (France, Ireland, the Netherlands and the UK). The trial is single-blinded. Participants, practitioners and the researcher conducting the qualitative research will know the arm to which patients are allocated; however, the statisticians conducting the quantitative analysis will be blinded to study allocation. While it has been powered to detect a small effect size (see below for details) in relation to the primary outcome (i.e. reduction in overall dementia risk modification score, which will be indicated by changes in participants’ LIBRA score before and after the trial), the principal aim is to test the feasibility and workability of the approach in routine primary care. This will be assessed through the use of qualitative methods, underpinned by the theoretical approach of normalisation process theory.

The design of this protocol has followed the recommendations of the SPIRIT guidelines [51, 52]. A full copy of the SPIRIT protocol can be found in Additional file 1.

### Trial registration and ethical approval

The In-MINDD RCT is registered with the ISRCTN Registry: ISRCTN 98553005 (DOI: 10.1186/ISRCTN98553005). Ethical approval has been obtained in each country in which the RCT will be conducted, namely France, Ireland, the Netherlands and the UK.

### Recruitment

First, 6–10 general practices will be recruited in each country; within each practice, up to 25 participants will be recruited, giving 150 research participants per country and a total study population of 600 patients across the four countries. General practices will be contacted through the existing links and networks of the in-country research teams and selected on their interest in taking part in the study but there will be attempts made to ensure a spread in terms of the socioeconomic status of the practice populations.

Part of the aim of this feasibility study is to collect data which will allow the calculation of a definitive sample size for an effectiveness RCT. However, we did calculate that a sample size of 600 participants will have power (0.8) to detect a small effect size (of 0.187 or 0.2). This lead us to the conclusion that 150 patients per partner country, randomised into either the In-MINDD group or a control group, will be sufficient to confidently show evidence of the effect over the timescale of the randomised controlled trial.

Participants will be identified from practice lists by either the research team or practice staff in Ireland, The Netherlands and France and by a recognised proxy in Scotland (the Scottish Primary Care Research Network). Once eligible patients have been identified, GPs will screen the list to ensure that only suitable participants are approached. The researcher or practice, as appropriate, will then write to eligible patients to ask if they are interested in participating in In-MINDD. In some participating practices, the study will also be advertised to patients through practice posters. If recruitment proves difficult through practices, ethical permission will be obtained to advertise and recruit out with general practices.

Potential participants will be sent an information pack about In-MINDD, including participant information sheets and an expression of interest form, which is returned to the research team. Those who will respond will be contacted by the research team and invited to meet with an In-MINDD researcher. At that meeting, eligibility will be confirmed and consent to participate in the study will be obtained. We will assume a response rate of 10–20 % (based on recent experience of recruitment for similar studies) for our initial mailing and will repeat mailings until our sample size has been achieved.

### Inclusion and exclusion criteria

Inclusion and exclusion criteria are described in Table 3.

**Table 3:** In-MINDD inclusion and exclusion criteria

Inclusion criteria
Registered with a participating practice
Age 40–60 on date of consent
Presence of any one (or more) of the following risk factors:
Depression—previous history OR active episode of minor depression as recorded on medical record (if GP deems patient fit to participate)
Diabetes (diagnosis, e.g. on a diabetes disease register)
Hypertension (as per national guidelines)
Renal dysfunction (recorded by GP)
Obesity (BMI of 30.0 or above)
Current smoker
Raised cholesterol (as per national guidelines)
Coronary heart disease (diagnosis, e.g. on a CHD disease register)
Self-reported sedentary lifestyle
Self-reported lack of cognitive stimulation
Medically stable
Literate in language of the partner country where patient is recruited
Access to the internet in order to communicate by email and access information online
Exclusion criteria
Active episode of major depression recorded in medical record or assessed using a validated assessment score, e.g. Hospital Anxiety and Depression Scale (HADS), and which GP deems makes patient too severely ill to participate
Unable to give informed consent
Has an existing diagnosis of dementia
Other reason identified by GP, e.g. terminally ill

### Baseline data collection

Baseline data will be collected from all participants using the In-MINDD on-line profiler; clinical data will be provided by his/her general practitioner. Data will be collected on the following variables:

- Background information: age, sex, marital status, employment status, education attainment, level of occupational attainment, and living arrangements,
- General health information,
- Family medical history (i.e. existence of dementia, cardiovascular disease and/or diabetes mellitus),
- Alcohol consumption,
- Current and past smoking habits,
- Blood pressure (obtained from general practice),
- Cholesterol level (obtained from general practice),
- Verification of diagnosis of cardiovascular disease, renal dysfunction and diabetes (obtained from general practice),
- General mental health and mood using the Center for Epidemiologic Studies Depression Scale (CESD), a short 20-item self-report scale designed to measure symptoms associated with depression in the general population [53],
- Physical activity using the European Prospective Investigation into Cancer and Nutrition (EPIC) physical activity questionnaire, which assesses physical activity in current occupation and in leisure and household domains in a typical week over the past year [54],
- Cognitive activity using the Adapted Cognitive Reserve Index questionnaire (CRIqadapted), adapted with permission from a short instrument developed in Italy by Nucci et al. [55] that assesses formal and non-formal education, occupational activity and frequency of participation in leisure time activities over an individual’s adult life (i.e. since the age of 18),
- Diet using the Mediterranean diet adherence screener (MEDAS), a brief dietary assessment instrument that was developed in Spain for the PREDIMED trial [56]. This 14-item instrument measures adherence to a Mediterranean diet enhanced with olive oil and nuts. Some minor adaptations have been made to the MEDAS instruments to make it suitable for use in non-Mediterranean countries such as Ireland and Scotland.

While self-reported measures can be subjected to bias in reporting, the aim of In-MINDD is to develop a scalable intervention which individuals can complete on-line. Therefore, scores obtained for measures of mental health and mood, physical activity, cognitive activity and diet, as well as alcohol consumption and smoking will not be verified with participants’ GPs*.* A number of the measures, e.g. the EPIC PAQ and Cambridge Index for physical activity, have been shown to correlate well with self-reported measures. In addition, with the exception of smoking and possibly alcohol consumption, it is unlikely that GPs will routinely collect data on the other variables of interest. Clinical data, such as blood pressure, cholesterol, and diagnosis of cardiovascular disease, renal dysfunction and diabetes, will be checked with GPs. Where a discrepancy is identified (e.g. the participants say that they do not have CVD, but their GP says they do), both the participants and GP, with the participant encouraged to seek an appointment with their GP to discuss this.

**Fig. 2:**
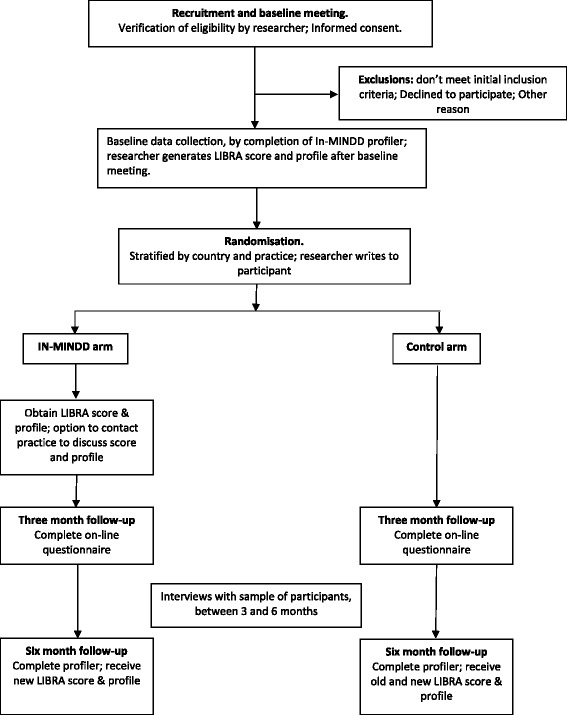
Flow diagram of In-MINDD RCT

Once the profiler has been completed, participants will be randomised to either the In-MINDD or control arm of the trial (Fig. 2).

### Randomisation

Participants will be randomised in equal proportions to either (i) the In-MINDD arm or (ii) the control arm of the trial. Randomisation will be conducted by the Robertson Centre for Biostatistics, University of Glasgow and will be stratified by country and by practice. The allocation will be derived using a computer-generated randomisation schedule following completion of the on-line profiler. The research team will then write to the patient to inform them of their allocation to either the In-MINDD or control arm of the trial.

### Intervention arm

Participants will receive information in form of a personalised lifestyle for brain health (LIBRA) global score and profile based on the demographic, health behaviour and clinical information entered into the In-MINDD profiler. This personalised profile will highlight those areas where they are doing well in terms of protecting their brain health, those areas where they can make sustainable lifestyle behavioural changes and conditions that they need to manage well (Fig. 1). Participants will have an opportunity, if they want, to discuss their LIBRA score, profile and personalised plan with their GP or other member of the general practice team, such as a practice nurse—either face-to-face or by telephone and will also be given access to the In-MINDD on-line support environment. This will allow them to access information on health-related behaviours, for example advice on healthy eating, advice on physical exercise, and links to smoking cessation services. The In-MINDD on-line support environment also provides links to existing national websites including, where possible, information on local services. So, for example, if someone decides that they wish to increase their physical activity, they will be able to access information about what is available locally on relevant websites accessed through the support environment.

Finally, the support environment incorporates goal setting, again is personalised for each individual according to their LIBRA profile. Participants will be able to set specific goals and will be able to self-monitor progress. They will supported by monthly email prompts regarding goal setting and attainment. Goal attainment will be assessed at the end of the 6-month trial.

At 3 months, participants will be asked to complete a short on-line questionnaire. This will ask about the LIBRA profile information they received, whether they have accessed the on-line support environment, whether they set any health behaviour change goals, and if yes, whether they have maintained that change. They will also be asked if they would be willing to be interviewed about their experience so far in In-MINDD. In addition, the use of the In-MINDD support environment will be monitored remotely, e.g. how often they access the systems, for how long, and what pages they visit.

After 6 months, participants will either meet with the researcher or self-complete and update their information on the In-MINDD system in order to generate a final LIBRA score and profile.

### Control arm

Participants in the control arm will receive generic health information material, e.g. on smoking cessation and increasing physical activity. Participants in the control arm will be asked to complete an on-line questionnaire at 3 months. They will be informed that we will contact them towards the end of the trial period, to identify anyone who may wish to be interviewed. At the end of the trial period, they will either meet with the researcher or self-complete the profiler. They will receive a copy of their LIBRA score and profile and will be given access to the In-MINDD on-line support environment.

### Process evaluation

A process evaluation will be conducted as part of the trial, in order to understand the experience of participants and practitioners using the In-MINDD profiler and on-line environment. A purposive sample of participants approaching the end of the trial period will be asked if they are willing to participate in a face-to-face interview. At the end of the trial, a sample of participants will be selected from those agreeing to be approached for interview or focus group. A minimum of five patients will be interviewed in each country (more in Ireland and Scotland, where interviews can be conducted in English and not need to be translated for analysis); interviewees will be selected on the basis of LIBRA score (high and low), gender (male and female). Interviews will be conducted at a time and place convenient to the interviewee and will last approximately 1 h. Interviews will explore their views of the information they received from the profiler, their use of the on-line support environment, how they did/did not incorporate recommendations into their daily life, and their perception of the overall impact of the In-MINDD experience. A smaller group of participants in the control group (*n* = 10) will also be interviewed in order to ascertain whether the information they received increased their understanding of the modifiable risk factors for dementia and whether they made any lifestyle changes. Efforts will also be made to obtain an understanding of why participants did not complete the trial by including a sample of those who dropped out.

We will also interview a sample of health professionals from participating practices to understand their expectations and opinion about reducing dementia risk and the In-MINDD system. We will recruit GPs, practice nurses and other appropriate staff members from participating practices in Ireland and Scotland. Staff will be interviewed about their views of dementia, the use of risk scores and how such information is shared with patients, preventive strategies for dementia, and the In-MINDD profiler.

Data analysis will use recognised methods of qualitative analysis [57], underpinned by Normalisation Process Theory [46, 49, 50]. Analyses will be conducted both within country and by sharing anonymised transcripts between countries via video-conferenced data coding clinics.

### Improving adherence

We will monitor the use of the In-MINDD on-line support environment remotely, e.g. how often they access the systems, for how long and what pages they visit. Questionnaires at 3 and 6 months will assess what areas of lifestyle behavioural change participants targeted, e.g. smoking cessation, taking up a new hobby such as learning a language, and to what extent they have maintained that activity. Participants will be recruited through general practices; if contact is lost at either 3 or 6 months, we will be able to contact the practice to ascertain if the patient is still registered with the practice or if they have left. This will be documented by the local country team. We will not, however, remove patients from the trial if they do not use the In-MINDD on-line environment, as sustainability of use is a key research questions.

### Discontinuing or modifying the intervention

Participants will be able to leave the trial, if they wish to, at any time and for any reason. An end of trial form will be completed for all trial members, detailing the reason for leaving the trial, e.g. choosing to leave, illness, death, and loss to follow-up.

As this is a feasibility trial, we are particularly interested in monitoring the sustainability of use of the In-MINDD intervention. This is not a medical or pharmaceutical intervention, so we will not modify the delivery of the intervention but, instead, monitor how participants use and adapt the system to suit their own needs. In particular, we are interested to explore if giving participants information about modifiable risks for dementia prompts them to modify their daily activities.

While we do not plan to alter the In-MINDD intervention within this feasibility study, the data generated from the process evaluation will be used to develop and modify the in-line profiler and support environment for future use.

We have not planned to provide GPs with training into the area of dementia prevention, instead relying on their current knowledge of risk factors associated with the later development of dementia and, in particular, their knowledge on advising patients on strategies to initiate and support health-relate behaviour change, such as smoking cessation or exercise uptake. This approach was supported by those GPs who were consulted during earlier co-design of the on-line profiler. Participating GPs did, however, have access to the In-MINDD materials made available to the participants, and they had the opportunity to ask questions of the research team if required. The process evaluation will, however, explore their views of this and should provide valuable information to promote the design and delivery of future training.

### Outcomes

Primary outcome measure is change in dementia risk modification (LIBRA) score over the 6 months of the trial, calculated on the basis of a basket of individual risk factors identified by earlier work in In-MINDD [12]. These will include physical and cognitive activity, mood, presence or absence of diabetes, chronic kidney disease and/or cardiovascular disease, high cholesterol, adherence to a Mediterranean diet, smoking status, alcohol consumption, hypertension, and obesity. Secondary outcomes will be changes in individual risk factors.

### Analyses plan

Analyses will be conducted by the Robertson Centre for Biostatistics, University of Glasgow. The primary outcome—the dementia risk modification score—will be analysed using a linear regression model, with a binary term for intervention group, and adjusting for the baseline risk score and country. This model will be extended to investigate baseline predictors of outcome, with interaction terms added to assess subgroup differences (e.g. age) in any intervention effect. Similar methods will be applied to individual risk factors. Modelling assumptions will be assessed through examination of residual distributions, and data transformations or generalised linear models will be used where appropriate. All analyses will be by intention to treat, i.e. in relation to randomised allocation, regardless of adherence to or uptake of the intervention. Multiple imputations will be used for any missing baseline information. Missing outcome data will not be imputed in the first instance, but the sensitivity of results to alternative assumptions will be assessed. Where appropriate, data will be presented with 95 % confidence intervals.

### Feasibility outcomes

A key part of this RCT will be to determine a set of feasibility outcomes to inform the development of a full-scale effectiveness trial. Feasibility outcomes are those outcomes which relate to the successful implementation and completion of the trial, including the route and ease of recruitment, ability of participants to enter and remain in the trial, and the ability of participants to complete the profiler.

### Parameters on which feasibility will be judged

Our principle parameters to determine the success of our feasibility study are:

1. Ease of recruitment—can we identify and recruit eligible patients in primary care, through general practices.
2. Successful completion of the profiler, with a loss to follow-up of no more than 20 % (i.e. completion rate of 80 % or more).
3. Evidence that the profiler is not harmful to patients, i.e. that there is no systematic worsening of LIBRA score in the intervention group compared to controls over the time period.

### Monitoring and patient safety

Monitoring of trial recruitment will be conducted by each partner country, using a trial timetable template. Trial coordinators in each country will communicate their progress monthly to the trial coordinators (SB and COD) in the University of Glasgow. Monitoring of the overall trial will be the responsibility of the trial monitoring committee. Serious adverse events (SAEs) are not anticipated during this trial, but unanticipated adverse events are always possible. Attempts will be made to monitor patients who are lost to follow-up or who drop out; practices will be asked about such patients in order to identify if the patient has experienced a harmful event (e.g. hospital admission, death) which could be attributed to In-MINDD. This will be recorded and sent to the trial monitoring committee.

## Discussion

Dementia is a growing challenge for health systems in the twenty-first century. There is, however, growing evidence that there are modifiable risk factors which can contribute to an individual’s risk of developing dementia in later life. In-MINDD has a key role to play both in raising awareness of this amongst the public and primary care practitioners and, through utilising the potential benefits of internet-based, personalised health prevention strategies and to alleviate the problem. Testing the intervention in a robust and theoretically informed manner will pave the way for a future, full-scale RCT.

## Trial status

Practice recruitment was initiated in June 2014. Patient recruitment began in October 2014 and is ongoing until June 2015. The trial will conclude in early 2016.

### Funding statement

In-MINDD is funded by the European Community’s Framework Programme Seven (FP7) under contract #304979. All materials developed as part of this study have been funded from this project.

Recruitment of practices and research participants in Scotland has been partly funded by the Chief Scientist Office Support for Science Funding, through the Scottish Primary Care Network (SPCRN) as part of its remit to support primary care-based research.

## Appendix 1

### Full In-MINDD team

Kate Irving, Maria Pierce, Muriel Redmond, Kevin Power, Lorraine Boran, Lisa McGarrigle.

School of Nursing and Human Sciences, Dublin City University, Dublin, Ireland.

Mark Roantree, Noel O’Kelly, Jim O’Donoghue, Alan Smeaton.

Insight Centre for Data Analytics, Dublin City University, Dublin, Ireland.

Catherine A O’Donnell, Susan Browne, Karen Woods.

General Practice and Primary Care, Institute of Health & Wellbeing, University of Glasgow, Glasgow, UK.

Alex McConnachie, Sarah Barry.

Robertson Centre for Biostatistics, Institute of Health & Wellbeing, University of Glasgow, Glasgow, UK.

Kay Deckers, Martin PJ van Boxtel, Sebastian Köhler, Marjan van den Akker, Marjolein de Vugt, Olga Schiepers, Stephanie Vos, Frans Verhey

Maastricht University, School for Mental Health and Neuroscience, Alzheimer Centrum Limburg, Maastricht, The Netherlands.

Valeria Manera, Jérémy Bourgeois, Anne Marie Cauvin, Philippe Robert

CoBTeK COgnition Behaviour Technology, Université de Nice Sophia Antipolis, Nice, France.

Nora Ward, Kylie O’Brien, Ciaran Clissman

Pintail Limited, Dublin Ireland.

## Additional file

Additional file 1: **In-MINDD protocol, following SPIRIT guidelines**. (DOCX 144 kb)
