# Supplementary material for: Reducing dementia risk by targeting modifiable risk factors in mid-life: study protocol for the Innovative Midlife Intervention for Dementia Deterrence (In-MINDD) randomised controlled feasibility trial
Source: Pilot Feasibility Stud. 2015 Nov 17;1:40. doi: 10.1186/s40814-015-0035-x (PMC5154057; doi:10.1186/s40814-015-0035-x)
Supplement: Additional file 1: — In-MINDD protocol, following SPIRIT guidelines. (DOCX 144 kb) [file 40814_2015_35_MOESM1_ESM.docx]

**Innovative Midlife Intervention for Dementia Deterrence (In-MINDD)**

**Protocol for the In-MINDD Feasibility Randomised Controlled Trial**

1. ***Title***

*In-MINDD*

Innovative Midlife Intervention for Dementia Deterrence (In-MINDD) Feasibility Randomised Controlled Trial: A multi-centre, primary care-based, investigator-blinded, randomised 6-month feasibility trial to compare the effectiveness of providing a dementia risk score – presented to patients as a Lifestyle for Brain Health (LIBRA) global score and profile - and access to internet-based self-help materials with routine practice for 40-60 year olds to reduce or delay the risk of developing dementia in later life.

1. ***Trial registration***

2a. ISRCTN 98553005 (DOI: 10.1186/ISRCTN98553005)

<http://www.controlled-trials.com/ISRCTN98553005/>

See Table 1 for full list of trial registration data.

1. ***Protocol version***

Issue date: 12 April 2015

Protocol Development v5.5

Authors: Catherine O’Donnell, Susan Browne.

1. ***Funding***

In-MINDD is funded by the European Community’s Framework Programme Seven (FP7) under contract #304979. All materials developed as part of this study have been funded from this project.

Recruitment of practices and research participants in Scotland has been partly funded by the Chief Scientist Office Support for Science Funding, through the Scottish Primary Care Network (SPCRN) as part of its remit to support primary care-based research.

Table 1. Trial registration data.

| Primary registry and trial identifier | ISRCTN  ISRCTN98553005 |
| --- | --- |
| Date of registration in primary registry | 7 May 2014 |
| Secondary identifying numbers | EU FP7 contract #304979 |
| Funder | European Community’s Framework 7 Programme |
| Primary sponsor | Sponsor required in UK only:  Dr Erica Packard  NHS Greater Glasgow and Clyde  Clinical Trials Unit  1^st^ Floor  Tennant Building  38 Church Street  Glasgow G11 6NT |
| Contact for public queries | Professor Kate O’Donnell  Kate.O’Donnell@glasgow.ac.uk |
| Contact for scientific queries | Professor Kate O’Donnell  Kate.O’Donnell@glasgow.ac.uk |
| Public title | Feasibility RCT: In-MINDD profiler and supportive environment |
| Scientific title | Innovative Midlife Intervention for Dementia Deterrence (In-MINDD) feasibility randomised controlled trial in four European primary care settings |
| Countries of recruitment | France, Ireland, The Netherlands, UK |
| Health condition(s) or problem(s) studied | Dementia |
| Intervention(s) | Intervention: Participants given personalised Lifestyle for Brain Health (LIBRA) score and profile based on their modifiable risk factors and access to In-MINDD on-line support environment. |
|  | Control: Generalised health information. Personalised Lifestyle for Brain Health (LIBRA) score and profile based on their modifiable risk factors and access to In-MINDD on-line support environment given to control group at end of study. |
| Key inclusion & exclusion criteria | Inclusion criteria:   - Registered with a participating practice - Age 40 – 60 on date of consent - Presence of any one (or more) of the following risk factors   - Depression – previous history OR active episode of minor depression as recorded on medical record – ***IF*** GP deems patient fit to participate   - Diabetes (diagnosis e.g. on a diabetes disease register)   - Hypertension (as per national guidelines)   - Obesity (BMI of 30.0 or above)   - Current smoker   - High cholesterol (as per national guidelines)   - Coronary heart disease (diagnosis e.g. on a CHD disease register)   - Self reported sedentary lifestyle   - Self reported lack of cognitive stimulation - Medically stable - Literate in language of the partner country where patient is recruited (English/Dutch/French as appropriate). - Access to the internet in order to communicate by email and access information online. |
|  | Exclusion criteria:   - Active episode of **major** depression, if GP deems patient too severely ill to participate, recorded in medical record or assessed using a validated assessment score e.g. HADS - People who are unable to give informed consent - People who have dementia |
| Study type | A multi-centre, primary care-based, investigator-blinded, randomised 6-month feasibility trial |
| Date of first enrolment | July 2014 |
| Target sample size | 150 per country; 600 in total |
| Recruitment status | Pending |
| Primary outcome | Global dementia risk score |
| Secondary outcomes | Change in individual risk factors |

1. ***Roles and responsibilities***

***5a. Authors’ contributions.***

Protocol development and drafting led by Catherine O’Donnell (COD) and Susan Browne (SB), General Practice & Primary Care, University of Glasgow.

Kate Irving (KI), School of Nursing and Human Science, Dublin City University conceived the overall In-MINDD study and is the project co-ordinator. The In-MINDD feasibility RCT (Randomised Control Trial) is one part of a larger programme of work which includes the construction and testing of a risk algorithm for the development of dementia; on-line risk factor profiler and internet-based support environment containing self-help materials and information to other support; and scientific and public dissemination of the work of In-MINDD.

Work package 1: Identification of a comprehensive model for dementia risk (led by Sebastian Köhler (SK), Martin van Boxtel (MvB) and Frans Verhey (VI), Maastricht University).

Work package 2: Development of the In-MINDD risk profiler & In-MINDD online support environment (led by Alan Smeaton (AS) and Mark Roantree (MR), School of Computing, Dublin City University).

Work package 3: Feasibility RCT to test effectiveness of In-MINDD system (led by Catherine O’Donnell (COD) and Alex McConnachie (AMcC), University of Glasgow).

Work package 4: Dissemination & exploitation (led by Ciaran Clissmann (CC), Pintail Ltd, Dublin).

***5b. Name and contact information for the trial sponsor***

Trial sponsorship is only required in Scotland – the trial sponsor is:

Dr Erica Packard

NHS Greater Glasgow and Clyde

Clinical Trials Unit

1^st^ Floor

Tennant Building

38 Church Street

Glasgow G11 6NT

***5c. Role of study sponsor and funders, if any, in study design; collection, management, analysis, and interpretation of data; writing of the report; and the decision to submit the report for publication, including whether they will have ultimate authority over any of these activities***

The funder has no role in the design of this study and will not have any role during its execution, analyses, interpretation of the data, or decision to submit results. The funder and the sponsor are responsible for auditing study data.

***5d. Composition, roles, and responsibilities of the coordinating centre, steering committee, endpoint adjudication committee, data management team, and other individuals or groups overseeing the trial, if applicable***

In-MINDD trial co-ordination centre is the University of Glasgow. The lead is Professor Catherine O’Donnell; the trial manager Dr Susan Browne, in her role as the principal researcher for work package 3. Statistical and data management support is provided by Dr Alex McConnachie, Robertson Centre for Biostatistics, University of Glasgow.

The trial steering committee will comprise COD, AMcC in Scotland. In addition, we will include Frances Mair (Professor of Primary Care Research (General Practice and Primary Care), who is part of the IN-MINDD Advisory Group and Ciaran Clissmann from Pintail. Professor Mair will chair the trial steering committee.

The trial steering committee will meet three times: once as the trial is preparing to commence; once around the trial mid-point; and once when data are being analysed. These meetings will be conducted by video conference. The purpose of the trial steering committee will be to comment on the design and execution of the trial and to ensure that the processes set in place for execution, analysis and presentation of the findings are robust and transparent. The trial steering committee will also monitor the trial for adverse events (see section 22 – Harms). The trial steering committee will be unblinded to intervention allocation. For that reason, we will not include representatives from the other partner countries. However, a report on trial progress will be made to the IN-MINDD team after each meeting.

1. ***Background and rationale***

***6a. Background:*** Dementia is a serious loss of cognitive ability beyond what might be expected from normal ageing. Currently incurable, the onset and development of dementia can create an enormous sense of insecurity within individuals and their families. Research has demonstrated high levels of anxiety amongst middle aged and young-old individuals about their memory, compounded by the fact that the debilitation associated with dementia makes it one of the most feared conditions in relation to ageing ^1,2^. At a system level, the ageing demographics of many countries means that caring and treating dementia is a growing global burden; in Europe the total cost of dementia care in 2005 was estimated to be €130 billion^3^. In the UK, dementia costs the economy 23 billion Euros per annum, more than cancer and heart disease combined^4^. In Ireland, the economic and social costs of dementia have been estimated to be €1.6 bn per annum.

A number of risk factors have been identified which can enhance or reduce one’s risk of developing dementia^2,5^. While some of the principal risk factors are non-modifiable, such as age and genetic factors, a surprising number are modifiable. These include hypertension; cholesterol; obesity; alcohol consumption; smoking; and levels of physical and cognitive activity. Thus, a key message underpinning the In-MINDD study is that there are steps that individuals can take in mid-life to mitigate their potential risk of developing dementia in later life. This, however, means identifying effective ways of supporting individuals to make – and maintain – changes in health-related behaviours. This is known to be a challenging task. Utilising the power of the internet as a social support, In-MINDD seeks to test the provision of information on dementia risk coupled to access to an on-line support environment with patients in four European primary care systems.

The overall aims of In-MINDD are to: (i) develop a robust dementia risk model based on modifiable risk factors; (ii) implement this risk model in collaboration with patients and practitioners, by designing and supporting an on-line user environment; and (iii) test the feasibility and effectiveness of the In-MINDD system through a primary care-based feasibility randomised controlled trial.

Specific objectives are:

1. To formulate and validate a multi-factorial model of dementia risk using a combination of Delphi approaches and data mining of retrospective datasets;
2. To use this risk algorithm to produce a personalised risk reduction strategy, reflecting the patient’s risk profile, for endorsed by a GP or other primary care practitioner. This will be supported by an on-line environment giving the patient access to information and self-help materials.
3. To validate this approach on two levels: in terms of its usability and value to primary care clinicians’ and to assess patient adherence to the risk reduction strategy.
4. To publicise the modifiable nature of lifestyle risk factors for dementia.
5. To encourage dissemination to the public of the individual’s capability to modify his or her own risk of dementia through incorporation of this message into policy and health promotion campaigns.

In order to understand how the In-MINDD risk profiler and online support environment are used and the work required by both individuals and practitioners to embed this into everyday life and practice, the study will be underpinned by the use of Normalisation Process Theory ^6-8^. This is a sociological theory which helps us to understand how complex interventions become embedded and routinised into everyday practice (or not) and the work that individuals, both patients and health care professionals, have to do in order to sustain that activity.

***6b. Choice of comparators.***

Patients in the In-MINDD arm of the feasibility study will complete the on-line profiler which gather s information on their clinical history and on behavioural factors identified in WP1 as contributing, or alleviating, dementia risk e.g. mid-life obesity, smoking, cognitive activity. These data calculate a individual’s dementia risk score. Given the potential sensitivity and negativity associated with a dementia risk score, participants will receive this information in form of a personalised Lifestyle for Brain Health (LIBRA) score and profile (see Appendix)^[[1]](#footnote-1)^. This is a personalised profile highlighting those areas where they are doing well in terms of protecting their brain health and those areas where they can make sustainable lifestyle behavioural changes. Patients will have an opportunity to discuss their LIBRA score and profile and personalised plan with a GP or other member of the general practice team, such as a practice nurse – either face-to-face or by telephone. Following this consultation, they will also be given access to the In-MINDD on-line support environment. This will allow them to access information on health-related behaviours, for example advice on health eating; advice on physical exercise; links to smoking cessation services.

Where possible, there will also be some information on local services. So, for example, if someone decides that they wish to reduce their risk of developing dementia by increasing their physical activity or taking up an activity such a learning a language, they will be able to access information about available classes through the In-MINDD support environment. Since several lines of evidence indicate that setting goals is an important strategy for helping people to make lifestyle changes, the support environment also incorporates goal setting, which again is personalised for each individual according to their LIBRA profile. Participants will be able to set specific goals and will be able to self-monitor progress. They will supported by regular prompts and feedback regarding goal attainment. Goal attainment will be tracked by the system. Other on-line supports include an Ask the Experts feature and may include a closed forum on Facebook.

Patients randomised to the control arm will be given generic health information about health-related behaviour change e.g. in relation to smoking cessation, increasing physical activity. At the end of the 6-month feasibility trial period, they will be given their LIBRA profile and access to the In-MINDD environment. These patients will be free to discuss this information with their GP or practice nurse, if they wish.

1. ***Objectives***

The overall objectives for In-MINDD are noted in Section 6a.

The specific objectives for the feasibility RCT are:

1. To compare the effectiveness of the In-MINDD intervention compared with care as usual in reducing the overall risk of developing dementia and on individual risk factors.
2. To explore how patients use the In-MINDD intervention in terms of access to and time spent on the on-line environment.
3. To explore the feasibility of using the In-MINDD risk profiler and on-line support environment from the perspective of patients.
4. To explore patients understanding of, attitudes towards and experiences of obtaining their Lifestyle for Brain Health score and profile.
5. To explore the supports and barriers to embedding behaviour changes into everyday life, and whether or not the In-MINDD intervention helps or hinders patients make and maintain changes to individual health-related behaviours.
6. To explore the feasibility of using the In-MINDD risk profiler from the perspective of primary care practitioners.
7. To understand practitioner views of the utility of the Lifestyle for Brain Health score and profile and how they relate that information to patients.
8. ***Trial design***

In-MINDD is a feasibility randomised controlled trial. The trial is single-blinded; patients, practitioners and the researcher conducting qualitative research will know the arm to which patients are allocated, however the statisticians conducting the quantitative analysis will be blinded to study allocation. While it has been powered to detect a small effect size in relation to the primary outcome (i.e. reduction in overall dementia risk score, which will be indicated by changes in their LIBRA score/profile), the principal aim is to test the feasibility and workability of the approach in routine primary care. This will be assessed through the use of qualitative methods, underpinned by the theoretical approach of Normalisation Process Theory.

1. ***Study setting***

The In-MINDD feasibility RCT will be based in four different primary care systems in Europe, namely: Ireland; The Netherlands; France; and Scotland. The lead organisations in each country are:

Ireland – Dublin City University (DCU)

The Netherlands - Maastricht University (MU)

France - Université Nice Sophia Antipolis (UNS)

Scotland – University of Glasgow (GU)

Within each country, the work will be located in general practice/family practices. A total of 6 - 10 general practices will be recruited in each country; within each practice, up to 25 patients will be recruited, giving 150 research participants per country and a total study population of 600 patients across the four countries. General practices will be selected on their interest in taking part in the study.

1. ***Eligibility criteria***

**Inclusion criteria**

- Registered with a participating practice
- Age 40 – 60 on date of consent
- Presence of any one (or more) of the following risk factors
  - Depression – previous history OR active episode of minor depression as recorded on medical record – ***IF*** GP deems patient fit to participate
  - Diabetes (diagnosis e.g. on a diabetes disease register)
  - Hypertension (as per national guidelines)
  - Obesity (BMI of 30.0 or above)
  - Current smoker
  - High cholesterol (as per national guidelines)
  - Coronary heart disease (diagnosis e.g. on a CHD disease register)
  - Self reported sedentary lifestyle
  - Self reported lack of cognitive stimulation
- Medically stable
- Literate in language of the partner country where patient is recruited (English/Dutch/French as appropriate).
- Access to the internet in order to communicate by email and access information online.

**Exclusion criteria**

- Active episode of **major** depression, if GP deems patient too severely ill to participate, recorded in medical record or assessed using a validated assessment score e.g. HADS
- People who are unable to give informed consent
- People who have dementia

**Assessment of eligibility.**

Research teams, practice staff or a proxy (e.g. the Scottish Primary Care Research Network) will review general practice medical records using the above inclusion and exclusion criteria to identify potentially eligible patients and invite them to take part in In-MINDD. This process is illustrated in Figure 1.

As an alternative route of recruitment, practices will display flyers describing the In-MINDD feasibility study. Patients who believe they are eligible can then ask their GP or practice nurse for more information about the study. However, they must then meet the eligibility criteria to be considered for the study.

If recruitment through practices proves difficult, then ethical approval will be sought to recruit participants directly.

**Figure 1. Identification of eligible patients and randomisation into In-MINDD.**

**Assessment of eligibility.**

1: Conducted in the practice based on age; medical stability and presence of one or more IN-MINDD risk factors which are readily available to practices e.g. age band and smoker; age band, diabetic and smoker.

In Scotland: Practice with support of SPCRN to invite patient to participate.

France, Ireland, theNetherlands: Practice or research team to invite patient to participate.

Or

2: Responds to poster/flyer in GP practice, discusses with GP or practice nurse and then contacts researcher to discuss eligibility.

**Excluded.**

Don’t meet initial inclusion criteria

Declined to participate

Other reason

**IN-MINDD arm**

**Control arm**

**Randomisation.**

Stratified by country and by practice.

**Excluded.**

Don’t meet initial inclusion criteria

Declined to participate

Other reason

**Recruitment and baseline meeting.**

Verification of eligibility by researcher.

Informed consent.

Baseline data collection, by completion of IN-MINDD profiler.

1. ***Interventions***

The In-MINDD CONSORT diagram is shown in Section 13.

***11a. Interventions for each group.***

**IN-MINDD baseline data collection meeting.**

All participants expressing interest in the study will receive an information pack about the study after which they can contact the IN-MINDD researcher, who will then arrange a baseline meeting. At this meeting, the researcher will inform them about the trial and review their eligibility to participate. If the patient is eligible and is willing to proceed, informed consent will be obtained.

Participants will be assigned a unique log-in to the In-MINDD site, which they should use each time they access the on-line environment. Access to the support environment will be blocked until after randomisation, ensuring that only those randomised to the In-MINDD intervention can access the support environment.

Following consent, the researcher will go through the baseline data collection with the patient using the IN-MINDD risk profiler.

Data on variables identified as risk factors but not held routinely in GP records (e.g. cognitive activity; physical ability) will be collected at this meeting.

**Baseline data collection**

Baseline data will be collected using the In-MINDD profiler. This has been purposely designed and developed for the In-MINDD project by the project team. The data collected via the profiler includes the following:

• Background information about participants (such as age, sex, marital status, employment status, education attainment, level of occupational attainment, and living arrangements),

• Information about the participant’s health.

• Information about family medical history (i.e. dementia, cardiovascular disease and diabetes mellitus)

• Information about alcohol consumption and current and past smoking habits

While most of this will be self-completed, some clinical data (e.g. blood pressure, cholesterol level, verification of diagnosis of cardiovascular disease and diabetes), will be provided by the GP using a short pro-forma. These clinical data will either be given by the GP to the research participants who will input the data themselves (e.g. the process in Ireland) or be sent by the GP to the research team who will input the data after the baseline meeting (e.g. the process in Scotland). Patients will be informed of this at the baseline meeting.

The In-MINDD profiler will also collect data on participants’ mood, physical activity, cognitive activity and diet via four validated instruments, which have been carefully selected and adapted where necessary.

The four instruments are:

• Center for Epidemiologic Studies Depression Scale (CESD), a well known and widely used short 20-item self-report scale, created by Radloff^9^, and designed to measure symptoms associated with depression in the general population;

• European Prospective Investigation into Cancer and Nutrition (EPIC) Physical activity questionnaire, a self-administered questionnaire, which assesses physical activity in current occupation and in leisure and household domains in a typical week over the past year^10^;

• Adapted Cognitive Reserve Index questionnaire (CRIqadapted), a short instrument developed in Italy by Nucci et al^11^. It is based on a literature review of the most relevant sources from the cognitive reserve literature and is a compound measure of formal and non-formal education, occupational activity and frequency of participation in leisure time activities over an individual’s adult life i.e. since the age of 18);

• MEditerranean Diet Adherence Screener (MEDAS), a brief dietary assessment instrument that was developed in Spain for the PREDIMED trial^12^. It is a 14-item instrument that measures adherence to a Mediterranean diet enhanced with olive oil and nuts. Some minor adaptations have been made to the MEDAS instruments to make it suitable for use in non-Mediterranean countries such as Ireland and Scotland. The MEDAS instrument has been adapted for use online.

Participants will be asked to complete the questions in the profiler, which takes approximately 15 to 20 minutes.

Once the profiler has been completed, participants will be randomised to either the IN-MINDD or control arm of the trial.

At the baseline meeting (and before randomisation), research participants may also be invited to complete a Stroop test. It is intended to use the Luminosity Stroop test, a web-based platform (permission pending) to measure response inhibition, which is an executive function. Performance on the Stroop task will not be used for computing the In-MINDD lifestyle for brain health score/profile and will not influence it. The purpose of including the Stoop test is exploratory. The aim is to examine the relationships between cognitive reserve, executive function and LIBRA score, since research suggests that EF is predictive of cognitive decline.

**Randomisation**

Participants will be randomised in equal proportions to either (i) the In-MINDD arm or (ii) the control arm of the trial. Randomisation will be conducted by the Robertson Centre for Biostatistics, University of Glasgow and will be stratified by country and by practice. Following completion of the profiler, the researcher will access the randomisation sequence either on-line or via telephone. The research team will then write to the patient to inform them of their allocation to either the In-MINDD or control arm of the trial.

**In-MINDD arm of trial.**

Participants in the In-MINDD arm will receive a letter telling them that they have been allocated to the In-MINDD arm of the trial and they should contact their GP or practice nurse and arrange an appointment, in order to discuss their Lifestyle for Brain Health (LIBRA) score and profile and personalised plan. This will be presented to participants as an overall risk score, in visual form, as well as a breakdown of the risk factors where they are doing well (e.g. alcohol consumption within recommended levels) and the risk factors where lifestyle changes are needed to improve their overall LIBRA score, e.g. smoking and lack of physical activity (see Appendix xx). This letter will also give them a link to the In-MINDD system, where they will be able to access their on-line LIBRA profile and the In-MINDD on-line support environment.

Participants will be asked to attend their general practice for an appointment with either the GP or practice nurse, who will talk them through their profiler result and personalised plan. If this is problematic, for example in Ireland a GP consultation typically costs patients €50-60, participants will be sent the information and a telephone consultation offered instead or participants can choose not to discuss the LIBRA score and profile with their primary care providers.

Participants will then be free to access and use information held in the In-MINDD online support environment. Examples of activities that might be suggested include making changes to diet to aid weight reduction, or increasing the amount of physical activity done; or taking up a new activity, such as learning a language. Participants will be able to access advice and support about these activities from the In-MINDD online system and invited to set goals for health-related behaviour change. During this time, they will receive email reminders about their targets and goals and advice offered about progress.

At three months, participants will be asked to complete an on-line questionnaire about risk factor(s) they have selected and the steps and actions they have taken, if any, to reduce that or those risk factors. This questionnaire will address risk factors they selected, what they have learned about reducing that risk factor, what steps/actions they took to follow the advice given, how they have accessed those activities and how often and whether they are still participating in those activities. They will also be asked if they would be willing to be interviewed about their experience so far in In-MINDD. In addition, use of the In-MINDD support environment will be monitored remotely, e.g. how often they access the systems; for how long; and what sites they visit.

After six months participants will meet with the researcher or practice nurse again and update their information on the In-MINDD system in order to generate a final LIBRA score and profile.

***Control arm of the trial.***

Participants in the control arm will receive a letter from the researcher telling them they are in the control arm; they will receive generic health information material e.g. on smoking cessation, increasing physical activity. Participants in the control arm will be asked to complete an on-line questionnaire at three months. (In order to maximise follow up rates participants who don’t complete the on-line questionnaire will be offered a telephone alternative.) They will be informed that we will contact them towards the end of the trial period, to identify anyone who may wish to be interviewed. At the end of the trial period they will meet again with the researcher and complete the profiler. They will receive a copy of their LIBRA score and profile and will be given access to the In-MINDD on-line support environment.

***Process evaluation.***

A process evaluation, focussed on patients, will be conducted in each partner country. Additional work, interviewing more patients and practitioners, will be conducted in Scotland and Ireland.

As participants approach the end of the trial period, we will contact them and ask if they would be willing to participate in a face-to-face interview. At the end of the trial a sample of participants will be selected from those agreeing to be approached for interview or focus group. A minimum of 12 patients will be interviewed in each country; interviewees will be selected on the basis of LIBRA score (high and low); gender (male and female). Interviews will be conducted at a time and place convenient to the interviewee and will last approximately 1 hour. This work will to explore their views of the information they received from the profiler, their use of the on-line support environment, and how they did/did not incorporate recommendations into their daily life. A discussion of their perception of the overall benefit of the In-MINDD experience will also take place.

A larger process evaluation will be conducted in Scotland, where interviews will be conducted with 20 to 25 participants. A smaller group of participants in the control group (n=10) will also be interviewed in order to ascertain whether the information they received increased their understanding of the modifiable risk factors for dementia and whether they made any lifestyle changes. Efforts will also be made to obtain an understanding of why participants did not complete the trial by including a sample of those who dropped out.

We also intend to explore health professionals’ expectations and opinion about reducing dementia risk and the In-MINDD system. We will do this through both semi-structured interviews and focus groups. We will ask a minimum of three health professionals (GPS, practice nurses and other appropriate staff members) from each of the six participating practices to contribute to this work which will take place at the beginning of the trial and again at the end of the trial. Staff will be interviewed about their views of dementia; the use of risk scores and how such information is shared with patients; preventive strategies for dementia; and the In-MINDD profiler.

Written consent will be obtained from both patients and professionals prior to interview or focus group. Interviews and focus groups will be recorded and transcribed verbatim. Data analysis will use recognised methods of qualitative analysis ^13^, underpinned by Normalisation Process Theory ^6,8^. Analyses will be conducted both within country and by sharing anonymised transcripts between countries via video conferenced data coding clinics.

**Summary schedule of assessments**

| **In-MINDD arm of trial** | **Control arm of trial** |
| --- | --- |
| IN-MINDD baseline data collection meeting with researcher  (Eligibility checked, consent obtained, baseline data collected) – 1 hour | IN-MINDD baseline data collection meeting with researcher  (Eligibility checked, consent obtained, baseline data collected) – 1 hour |
| Participants receive letter informing which arm of the trial they are allocated to. | Participants receive letter informing which arm of the trial they are allocated to. |
| Participants access In-MINDD online support site and views LIBRA score. | Participants receive printed generic health information |
| Participants attend GP practice to discuss profiler result and personalised plan. |  |
| During this six month period participants are invited to access In-MINDD support environment and set goals for health related behaviour change. |  |
| At three months participants are asked to complete an online questionnaire | At three months participants are asked to complete an online questionnaire |
| After six months participants meet with researcher or practice nurse to update information and receive a final LIBRA score | After six months participants meet with researcher or practice nurse to update information and receive a final LIBRA score |

***11b. Discontinuing or modifying the intervention.***

Participants will be able to leave the trial, if they wish to, at any time and for any reason. An end of trial form will be completed for all trial members, detailing the reason for leaving the trial e.g. choosing to leave; illness; death; loss to follow-up.

As this is a feasibility trial, we are particularly interested in monitoring the sustainability of use of the In-MINDD intervention. This is not a medical or pharmaceutical intervention, so we will not modify the delivery of the intervention but, instead, monitor how participants use and adapt the system to suit their own needs. In particular, we are interested in exploring the actions taken by those in the In-MINDD arm in comparison with those in the control arm. For example, will the information about modifiable risk factors for dementia given to participants in the control arm prompt them to modify their lifestyle behaviour of their own volition (e.g. will simply knowing that smoking increases the risk of developing dementia encourage them to seek out information about smoking cessation and attempt to reduce their smoking or stop smoking completely; will knowing that obesity and physical inactivity increases the risk of developing dementia encourage them to take action to increase physical activity).

***11c. Improving adherence.***

We will monitor use of the In-MINDD on-line support environment remotely, e.g. how often they access the systems; for how long; and what sites they visit. Questionnaires at 3 and 6 months will assess what areas of lifestyle behavioural change participants targeted e.g. smoking cessation; taking up a new hobby such as learning a language, and to what extent they have maintained that activity. Participants will be recruited through general practices; if contact is lost at either 3 or 6 months, we will be able to contact the practice to ascertain if the patient is still registered with the practice or if they have left. This will be documented by the local country team. We will not, however, remove patients from the trial is they do not use the In-MINDD on-line environment, as likelihood and sustainability of use are key research questions.

***11d. Concomitant care.***

We will not actively prohibit other care interventions during the trial, but will ask about activities that may support or mitigate against In-MINDD. We will also interview participants randomised to the In-MINDD intervention arm; in Scotland, we will also interview control arm patients.

1. ***Outcomes***

Primary outcome: global risk score, calculated on the basis of a basket of individual risk factors identified from work package 1 of In-MINDD. These will include physical and cognitive activity; mood; presence of diabetes, chronic kidney disease and/or cardiovascular disease; high cholesterol; unhealthy diet; smoking status; alcohol consumption; hypertension; and obesity.

Secondary outcomes: changes in individual risk factors.

1. **Participant timeline**

**CONSORT Diagram.**

**Assessment of eligibility.**

Conducted in the practice based on age; medical stability and presence of one or more IN-MINDD risk factors which are readily available to practices e.g. age band and smoker; age band, diabetic and smoker.

Practice or research team to invite patient to participate.

**Control arm**

**IN-MINDD arm**

**Randomisation.**

Stratified by country and (possibly) by practice; researcher writes to participant

**2 Weeks**

Researcher adds in additional clinical information from practice; generates dementia risk score.

**Excluded.**

Don’t meet initial inclusion criteria; Declined to participate; Other reason

**Recruitment and baseline meeting.**

Verification of eligibility by researcher; Informed consent.

Baseline data collection, by completion of IN-MINDD profiler.

**Excluded.**

Don’t meet initial inclusion criteria; Declined to participate; Other reason

**1 month**

Visit practice or telephone consultation to obtain LIBRA score & profile; discussion

Interviews with sample of participants, between 3 and 6 months

**Six month follow-up**

Meet researcher; complete profiler; receive old and new LIBRA score & profile

**Six month follow-up**

Meet researcher; complete profiler; receive new LIBRA score & profile

**Three month follow-up**

Complete online questionnaire

**Three month follow-up**

Complete on-line questionnaire

**Control arm**

**IN-MINDD arm**

1. **Sample size**

Power calculations - Sample size = 600, power (.8) to detect a small effect size (.187 or.2) - indicate that 150 patients per partner country, randomised into either the In-MINDD group or a control group, will be sufficient to confidently show evidence of effect over the timescale of the randomised controlled trial.

1. **Recruitment**

Practice records will be screened on the basis of the inclusion criteria described in Section 10. Screening will be undertaken by either the research team or practice staff in Ireland, The Netherlands and France or by a recognised proxy in Scotland (Scottish Primary Care Research Network). Once eligible patients have been identified, GPs will screen the list to ensure that only suitable participants are approached. The researcher or practice, as appropriate, will then write to eligible patients to ask if they are interested in participating in IN-MINDD. (In Scotland, letters will be sent from participating practices with support of SPCRN).

Patients will be sent an information pack about In-MINDD, including participant information sheets and an expression of interest form, which is returned to the research team. Patients expressing an interest will be contacted by the research team and invited to meet with he researcher; at that meeting eligibility will be confirmed and consent to participate in the study will be obtained. We will assume a response rate of 10 - 20% (based on recent experience of recruitment for similar studies) for our initial mailing and we will repeat mailings until our sample size has been achieved.

1. **Allocation**

***16a. Sequence generation***

The Robertson Centre for Biostatistics, University of Glasgow will carry out the process of randomisation. Randomisation will be stratified by country and practice to ensure an even balance of participants across both arms of the trial, both within practices and between partner countries. The allocation will be derived using a computer generated randomisation schedule.

***16b. Concealment mechanism***

The randomisation sequence will be generated by the Robertson Centre for Biostatistics, University of Glasgow, who will not be involved in the face-to-face recruitment and consenting of participants. Allocation concealment will be ensured as randomisation will only occur once the patient has been consented into the study and baseline data have been collected. Research teams will allocate participants to arms of the trial using an online tool (telephone backup will be available).

***16c. Implementation of allocation***

The Robertson Centre will generate the allocation sequence. The research team in each partner country will enrol the patients into the trial. After the researcher has collected baseline data, they will assign patients to either the intervention or control arm of the trial by accessing the randomisation schedule either on-line or through use of a Smartphone app.

1. **Blinding**

***17a. Trial blinding***

The trial is single-blinded; patients, practitioners and the researcher conducting qualitative research will know the arm to which patients are allocated, however the statisticians conducting the quantitative analysis will be blinded to study allocation.

***17b. Emergency unblinding***

Emergency unblinding will be the responsibility of the trial steering committee. Emergency unblinding will only be permitted if there is evidence of intervention-related deaths, as a result of receiving a LIBRA score and profile and/or accessing the on-line support environment.

1. **Data collection methods**

Baseline data will be collected using the In-MINDD profiler. This has been purposely designed and developed for the In-MINDD project by the project team. The data collected via the profiler includes the following:

• Background information about participants (such as age, sex, marital status, employment status, education attainment, level of occupational attainment, and living arrangements),

• Information about the participant’s health.

• Information about family medical history (i.e. dementia, cardiovascular disease and diabetes mellitus)

• Information about alcohol consumption and current and past smoking habits

While most of this will be self-completed, some clinical data (e.g. blood pressure, cholesterol level, verification of diagnosis of cardiovascular disease and diabetes), will be provided by the GP using a short pro-forma. These clinical data will be inputted by the research team after the baseline meeting. Patients will be informed of this at the baseline meeting.

The In-MINDD profiler will also collect data on participants’ mood, physical activity, cognitive activity and diet via four validated instruments, which have been carefully selected and adapted where necessary.

The four instruments are:

• Center for Epidemiologic Studies Depression Scale (CESD), a well known and widely used short 20-item self-report scale, created by^9^, and designed to measure symptoms associated with depression in the general population;

• European Prospective Investigation into Cancer and Nutrition (EPIC) Physical activity questionnaire, a self-administered questionnaire, which assesses physical activity in current occupation and in leisure and household domains in a typical week over the past year^10^;

• Adapted Cognitive Reserve Index questionnaire (CRIqadapted), a short instrument developed in Italy by Nucci et al^11^. It is based on a literature review of the most relevant sources from the cognitive reserve literature and is a compound measure of formal and non-formal education, occupational activity and frequency of participation in leisure time activities over an individual’s adult life i.e. since the age of 18);

• MEditerranean Diet Adherence Screener (MEDAS), a brief dietary assessment instrument that was developed in Spain for the PREDIMED trial^12^. It is a 14-item instrument that measures adherence to a Mediterranean diet enhanced with olive oil and nuts. Some minor adaptations have been made to the MEDAS instruments to make it suitable for use in non-Mediterranean countries such as Ireland and Scotland. The MEDAS instrument has been adapted for use online.

Participants will be asked to complete the questions in the profiler, which takes approximately 15 to 20 minutes. This will be repeated at the end of the trial period.

At the baseline meeting (and before randomisation), research participants may also be invited to complete a Stroop test. It is intended to use the Luminosity Stroop test, a web-based platform (permission pending) to measure response inhibition, which is an executive function. Performance on the Stroop task will not be used for computing the In-MINDD lifestyle for brain health score/profile and will not influence it. The purpose of including the Stoop test is exploratory. The aim is to examine th3e relationships between cognitive reserve, executive function and LIBRA score, since research suggests that EF is predictive of cognitive decline.

1. **Data management**

Profiler data will be collect in each country by accessing a password protected site designed for In-MINDD by the computing scientists at Dublin City University. Data will be anonymised, with participants each allocated a unique study number. Data will be held securely through a Google App Engine cloud web application. Security is a key component of each of Google’s cloud computing elements, including Google App and other cloud web applications. For example, Google’s approach to IT security and the level of security guaranteed for the Google App engine are outlined a Google White Paper, which is available at the following link: https://cloud.google.com/files/GoogleCommonSecurityWhitePaperv1.4.pdf

Data will be transferred securely to the Robertson Centre for Biostatistics (RCB) in the University of Glasgow for analysis. The Centre sits in the Glasgow Clinical Trials Unit (GCTU), a United Kingdom Clinical Research Collaboration fully registered CTU. The source data will be stored on the RCB secure filestore and uploaded to the study database. Both the filestore and the study database will be backed up daily. Tapes will be stored in a fire-proof safe every two days and stored off-site every seven days. All data handled by the RCB will be anonymised and access restricted to study personnel.

The RCB manages all studies in accordance with its internal standard operating procedures and all relevant legal and regulatory guidelines. It has extensive experience of managing data in the context of UK and EU privacy and data protection legislation. The RCB is certified for ISO 9001:2008 for its quality systems, has TickIT accreditation for its software development and is BS7799 compliant.

1. **Statistical methods**

A detailed statistical analysis plan will be written and approved prior to the final unblinded analysis, according to RCB standard operating procedures. The primary outcome will be the global risk score, which will be analysed using a linear regression model, with a binary term for intervention group, and adjusting for the baseline risk score and country. This model will be extended to investigate baseline predictors of outcome, and interaction terms added to assess subgroup differences in any intervention effect. Similar methods will be applied to individual risk factors. Modelling assumptions will be assessed through examination of residual distributions, and data transformations or generalized linear models will be used where appropriate. All analyses will be by intention to treat, i.e. in relation to randomised allocation, regardless of adherence to or uptake of the intervention. Multiple imputation will be used for any missing baseline information. Missing outcome data will not be imputed in the first instance, but the sensitivity of results to alternative assumptions will be assessed.

1. **Monitoring**

Monitoring of trial recruitment will be conducted by each partner country, using a trial timetable template. Trial co-ordinators in each country will communicate their progress monthly to the trial co-ordinators (SB and KOD) in the University of Glasgow.

Monitoring of the overall trial will be the responsibility of the trial monitoring committee (see Section 5d).

1. **Harms**

Serious adverse events (SAEs) are not anticipated during this trial, but unanticipated adverse events are always possible. Attempts will be made to monitor patients who are lost to follow-up or who drop out; practices will be asked about such patients in order to identify if the patient has experienced a harmful event (e.g. hospital admission; death) which could be attributed to In-MINDD. This will be recorded and sent to the trial monitoring committee.

A serious adverse event (SAE) is defined as an untoward occurrence that:

(a) results in death,

(b) is life-threatening,

(c) requires hospitalisation or prolongation of existing hospitalisation,

(d) results in persistent or significant disability or incapacity,

(e) consists of a congenital anomaly or birth defect, or

(f) is otherwise considered medically significant by the investigator.

If a research participant experiences a serious adverse event (SAE) this will be reported to the Research Ethics Committee that gave a favourable opinion of the study (the 'main REC') and Sponsor where in the opinion of the Chief Investigator the event was:

- 'related': that is, it resulted from administration of any of the research procedures; and
- 'unexpected': that is, the type of event is not listed in the protocol as an expected occurrence.

Reports of related and unexpected SAEs will be submitted within 15 days of the Chief Investigator becoming aware of the event.

Additionally, attempts will be made to monitor patients who are lost to follow-up or who drop.

1. **Auditing**

The study may be audited randomly by local audit teams in each partner country. In Scotland the study may be audited by the trial sponsor.

1. **Research ethics approval - Plans for seeking REC/IRB approval**

Each partner country will apply for the relevant approvals in their country.

DCU:

- DCU Research Ethics Committee
- Irish College of General Practitioners Research Ethics Committee

*UM:*

- *Ethics Committee Maastricht University Medical Centre*

*UNS:*

- **CPP** sud-est (Comité de Protection des Personnes, *Commettee for human research protection*).
- **ANSM** (Agence National de sécurité du medicaments ; *French Agency for the Safety of Health Products*)
- **CCITRS** (Comité consultatif sur le traitement de l’information en matière de recherche dans le domaine de la santé, *Advisory committee on data processing in the domain of research on health*)
- **CNIL** (Comité National Informatique et Liberté, *National commettee for informatics and freedom*)

GU :

- *REC (Research Ethics Committee)*
- *NHS R&D Management Approval*

1. **Protocol amendments**

Any change in the study protocol will require an amendment. Any proposed protocol amendments will be initiated by the CI and any required amendment forms will be submitted to REC. The Sponsor will determine whether an amendment is non-substantial or substantial and will review all amended documents prior to submission to REC. Before the amended protocol can be implemented (or sent to other participating sites) favourable opinion/approval must be sought from the original reviewing REC and Sponsor.

1. **Consent or assent**

Experienced researchers will introduce the trial to potential participants who will be shown the participant information sheet and have the opportunity to ask questions. Researchers will obtain written consent from participants willing to participate in the trial. Information sheets and consent forms are provided for all participants in the trial.

1. **Confidentiality**

All study related information will be stored securely at each of the study sites. All participant information will be stored in locked filing cabinets. All participant information will be identified by a coded ID (identification) number to maintain participant confidentiality. All records containing names or other personal identifiers, such as consent forms, will be stored separately from study records identified by ID number, in locked filing cabinets.

1. **Declaration of interests**

There are no competing interests.

1. **Access to data**

Data access will be restricted to members of the In-MINDD team. All stored data will be anonymised; data will not be stored next to patient identifiers.

Data will be stored for 10 to 15 years depending on in-country requirements.

1. **Ancillary and post-trial care**

This is a feasibility trial in general practice. Participants will be free to seek whatever care they need or want, as normal, from their GPs and from other sources.

1. **Dissemination policy**

The results of the feasibility RCT will be reported to the European Commission as the final deliverable from the In-MINDD team.

Academic dissemination will include the publication of the trial protocol in a peer-reviewed open access journal, e.g. The Lancet or BMJ Open. Study outcomes will also be reported in a number of formats including peer-reviewed journal papers; reports to organisations working in the field of dementia, for example Alzheimer’s Scotland; and through presentations at academic and practitioner conferences.

Findings from the study will also be disseminated through the In-MINDD website http://www.inmindd.eu/

1. **Informed consent materials**

Consent form and other related documentation will be given to all participants – both patients and practitioners.

1. **Biological specimens**

There will be no biological specimens collected as part of In-MINDD.

**References**

1. Mol MEM, van Boxtel MPJ, Willems D, Verhey FRJ, Jolles J. Subjective forgetfulness is associated with lower quality of life in middle-aged and young-old individuals: A 9-year follow-up in older participants from the Maastricht Aging Study. *Aging & Mental Health* 2009; **13**(5): 699-705.

2. Desai AK, Grossberg GT, Chibnall JT. Healthy brain aging: A road map. *Clinics in Geriatric Medicine* 2010; **26**(1): 1-16.

3. Wimo A, M. P. World Alzheimer Report 2010. The global economic impact of dementia. Stockholm, 2010.

4. Luengo-Fernández R, Leal J, Gray A. Dementia 2010. The economic burden of dementia and associated research funding in the United Kingdom. Cambridge: Alzheimer's Research Trust, 2010.

5. Kloppenborg RP, van den Berg E, Kappelle LJ, Biessels GJ. Diabetes and other vascular risk factors for dementia: Which factor matters most? A systematic review. *European Journal of Pharmacology* 2008; **585**(1): 97-108.

6. May CR, Mair F, Finch T, et al. Development of a theory of implementation and integration: Normalization Process Theory. *Implementation Science* 2009; **4**(1): 29.

7. May C, Finch T. Implementing, embedding, and integrating practices: An outline of Normalization Process Theory. *Sociology* 2009; **43**(3): 535-54.

8. MacFarlane A, O'Donnell C, Mair F, et al. REsearch into implementation STrategies to support patients of different ORigins and language background in a variety of European primary care settings (RESTORE): study protocol. *Implementation Science* 2012; **7**: 111.

9. Radloff LS. The CES-D scale: a self-report depression scale for research in the general population. *Applied Psychological Measurement* 1977; **1**: 385-401.

10. Cust AE, Smith BJ, Chau J, et al. Validity and repeatability of the EPIC physical activity questionnaire: a validation study using accelerometers as an objective measure. *International Journal of Behavioral Nutrition and Physical Activity* 2008; **5**: 33.

11. Nucci M, Mapelli D, Mondini S. Cognitive Reserve Index questionnaire (CRIq): a new instrument for measuring cognitive reserve. *Aging, Clinical and Experimental Research* 2012; **24**(3): 218-26.

12. Martinez-Gonzalez MA, Garcia-Arellano A, Toledo E, et al. A 14-item Mediteranean diet assessment tool and obesity indexes among high-risk subjects: The PREDIMED trial. *PLoS ONE* 2012; **7**(8): e43134.

13. Ritchie J, Spencer L. Qualitative data analysis for applied policy research. In: Bryman A, Burgess RG, eds. Analyzing qualitative data. London: Routledge; 1994: 173-94.

1. In the LIBRA profile, an individual’s “room for improvement” score equates to their overall dementia risk score, expressed as a percentage. So, the greater their “room for improvement score”, the higher their potential chance of developing dementia in later life. [↑](#footnote-ref-1)
